# The Book World of Medicine and Science

**Published:** 1894-11-24

**Authors:** 


### THE BOOK WORLD OF MEDICINE AND SCIENCE.

SCIENCE PROGRESS. Vol. I. (The Scientific Press, London.)

We have received for review the first volume of "Science Progress" which contains the numbers appearing during the six months, March—August. The leading idea of the new journal is to provide a reliable source of information to which anyone engaged in scientific work can appeal in order to make himself aware of what is being done in kindred branches of science. No one can fail to recognise the value of such an undertaking, for it will be within the experience of many a worker that a fact picked up, it may be by chance, often serves to complete the elucidation of a difficult problem. It is a fact, however, that up to quite recently, although several scientific journals of high repute were in existence, there was no periodical devotod entirely to first-rate articles by well-known men on subjects altogether scientific. Each article in "Science Progress" aims at providing not merely an authoritative exposition of the subject-matter in hand, but also at supplying a really useful bibliography. Amongst the articles to which we would point as especially fulfilling the object of the promotors is one by Dr. Starling, entitled "Researches on Proteid Metabolism," which appeared in the second number, and which is a masterly review, compressed into a very few pages, of the important work recently carried out by Pflüger and his pupils upon one of the most interesting questions of the metabolism of the animal body. Another paper deserving of special mention, and likely to be of great importance from a commercial point of view, is entitled, "Pure Yeast, and its Relations to Brewing Operations," by Dr. A. K. Miller. Other articles which may be mentioned as particularly well fulfilling the object of the journal are, "The Most Recent Values of the Magnetic Elements at the Principal Magnetic Observatories of the World," by C. Chree, Superintendent of the Kew Observatory; "The Arrangement of the Molecules in a Crystal," by H. A. Miers; and "On the New Theory of Solutions," by J. W. Rodger. To the two last numbers in the volume are added a few pages containing an enumeration of titles of all the chemical papers which appeared during the previous month. As these pages are inserted as an appendix, it is obvious that no article matter has been sacrificed, and the possession of the titles in so compact a form will doubtless be a convenience to many workers. We feel no hesitation in saying that the professed objects of the promoters have been admirably carried out in the first volume. The journal thoroughly deserves to meet with warm support at home and abroad, and we hope to welcome it as a permanent addition to our scientific literature. It is, of course, always difficult for specialists to understand how abstruse the problems of their own science appear to others, and the risk undoubtedly exists of authors forgetting that they are writing for men with perhaps only a general acquaintance with the subject under discussion, and so of employing terms which to the general reader may be unintelligible. But this is a danger which may be guarded against without necessarily falling into the opposite extreme of superficiality. Evidently, no expense has been spared in the general get-up of the volume, the paper and printing being worthy of an *edition de luxe*, and altogether well adapted to withstand rough and constant usage.

